# Cross-Education of Muscular Endurance: A Scoping Review

**DOI:** 10.1007/s40279-024-02042-z

**Published:** 2024-05-17

**Authors:** Jun Seob Song, Yujiro Yamada, Ryo Kataoka, William B. Hammert, Anna Kang, Jeremy P. Loenneke

**Affiliations:** Department of Health, Exercise Science, and Recreation Management, Kevser Ermin Applied Physiology Laboratory, The University of Mississippi, P.O. Box 1848, University, MS 38677 USA

## Abstract

**Background:**

It is well established that performing unilateral resistance training can increase muscle strength not only in the trained limb but also in the contralateral untrained limb, which is widely known as the cross-education of strength. However, less attention has been paid to the question of whether performing unilateral resistance training can induce cross-education of muscular endurance, despite its significant role in both athletic performance and activities of daily living.

**Objectives:**

The objectives of this scoping review were to provide an overview of the existing literature on cross-education of muscular endurance, as well as discuss its potential underlying mechanisms and offer considerations for future research.

**Methods:**

A scoping review was conducted on the effects of unilateral resistance training on changes in muscular endurance in the contralateral untrained limb. This scoping review was conducted in PubMed, SPORTDiscus, and Scopus.

**Results:**

A total of 2000 articles were screened and 21 articles met the inclusion criteria. Among the 21 included studies, eight studies examined the cross-education of endurance via absolute (*n* = 6) or relative (*n* = 2) muscular endurance test, while five studies did not clearly indicate whether they examined absolute or relative muscular endurance. The remaining eight studies examined different types of muscular endurance measurements (e.g., time to task failure, total work, and fatigue index).

**Conclusion:**

The current body of the literature does not provide sufficient evidence to draw clear conclusions on whether the cross-education of muscular endurance is present. The cross-education of muscular endurance (if it exists) may be potentially driven by neural adaptations (via bilateral access and/or cross-activation models that lead to cross-education of strength) and increased tolerance to exercise-induced discomfort. However, the limited number of available randomized controlled trials and the lack of understanding of underlying mechanisms provide a rationale for future research.

## Key Points

**Table:** 

Performing unilateral resistance training can increase muscle strength not only in the trained limb but also in the contralateral untrained limb, which is known as the cross-education of strength. However, less attention has been paid to the question of whether performing unilateral resistance training can increase muscular endurance in the contralateral untrained limb (i.e., cross-education of muscular endurance).
The current body of the literature does not provide sufficient evidence to draw clear conclusions whether a cross-education of muscular endurance is present. Therefore, further research with a nonexercise control group (i.e., randomized controlled trials) is necessary to draw strong conclusions.
The cross-education of muscular endurance (if it exists) may be potentially driven by neural adaptations (via bilateral access and/or cross-activation models that lead to cross-education of strength) and increased tolerance to exercise-induced discomfort.

## Introduction

Resistance training leads to improvements in strength and muscular endurance [1–3]. When resistance training is performed on one side of the body only (i.e., unilateral resistance training), increased muscle strength has been observed not only in the trained limb but also in the contralateral untrained limb, which is widely known as the cross-education (or cross-transfer) of strength [4, 5]. The cross-education of strength was first reported in the scientific literature as early as the late nineteenth century [6], and thereafter it has been studied and reviewed extensively over the years [4, 5, 7–9]. Although its underlying mechanisms are not entirely understood, there is a general consensus within the cross-education literature that the transfer of strength to the untrained limb is mediated primarily by neural mechanisms and likely not by mechanisms at the local muscle level (e.g., changes in muscle fiber type and cross-sectional area) as these changes appear to occur within the trained limb only [4, 7, 10, 11]. In contrast to cross-education of strength, considerably less attention has been paid to the question of whether performing unilateral resistance training can increase muscular endurance in the contralateral untrained limb (i.e., cross-education of muscular endurance).

Muscular endurance refers to the ability of muscles to perform successive contractions at a submaximal load, and it is considered as an important physical fitness component not only for athletic performance in sports but also for activities of daily living that require repetitive work [12]. Muscular endurance can be further specified into absolute and relative muscular endurance [13]. Absolute muscular endurance involves performing a maximal number of repetitions with a given absolute load regardless of changes in maximal strength (e.g., using 60% of pretraining 1RM at pre- and posttesting) [14]. In contrast, relative muscular endurance involves an individual performing a maximal number of repetitions with a load corresponding to a specific relative intensity or percentage of the individual’s current 1RM (e.g., using 60% of pretraining and posttraining 1RM at pre- and posttesting, respectively) [14]. In addition, muscular endurance has been measured in several other ways when using different types of testing (e.g., isometric, isokinetic), such as time to task failure or total work during repeated isokinetic contractions [15, 16]. There is evidence that resistance training can increase strength as well as induce positive mitochondrial and microvascular adaptations (e.g., mitochondrial respiratory capacity, capillary to fiber ratio), which may help explain muscular endurance adaptations in the trained limb [17–19]. However, it remains unclear whether these mechanisms can also explain the changes in muscular endurance in the contralateral untrained limb. Therefore, the purpose of this paper was to provide an overview of the existing literature on cross-education of muscular endurance following unilateral resistance training and to discuss its potential underlying mechanisms.

## Methods

A scoping review was conducted to evaluate the cross-education of muscular endurance. The current study was conducted and reported in accordance with the Preferred Reporting for Systematic Reviews and Meta-Analyses extension for scoping reviews (PRISMA-ScR) [20].

**Fig. 1:**
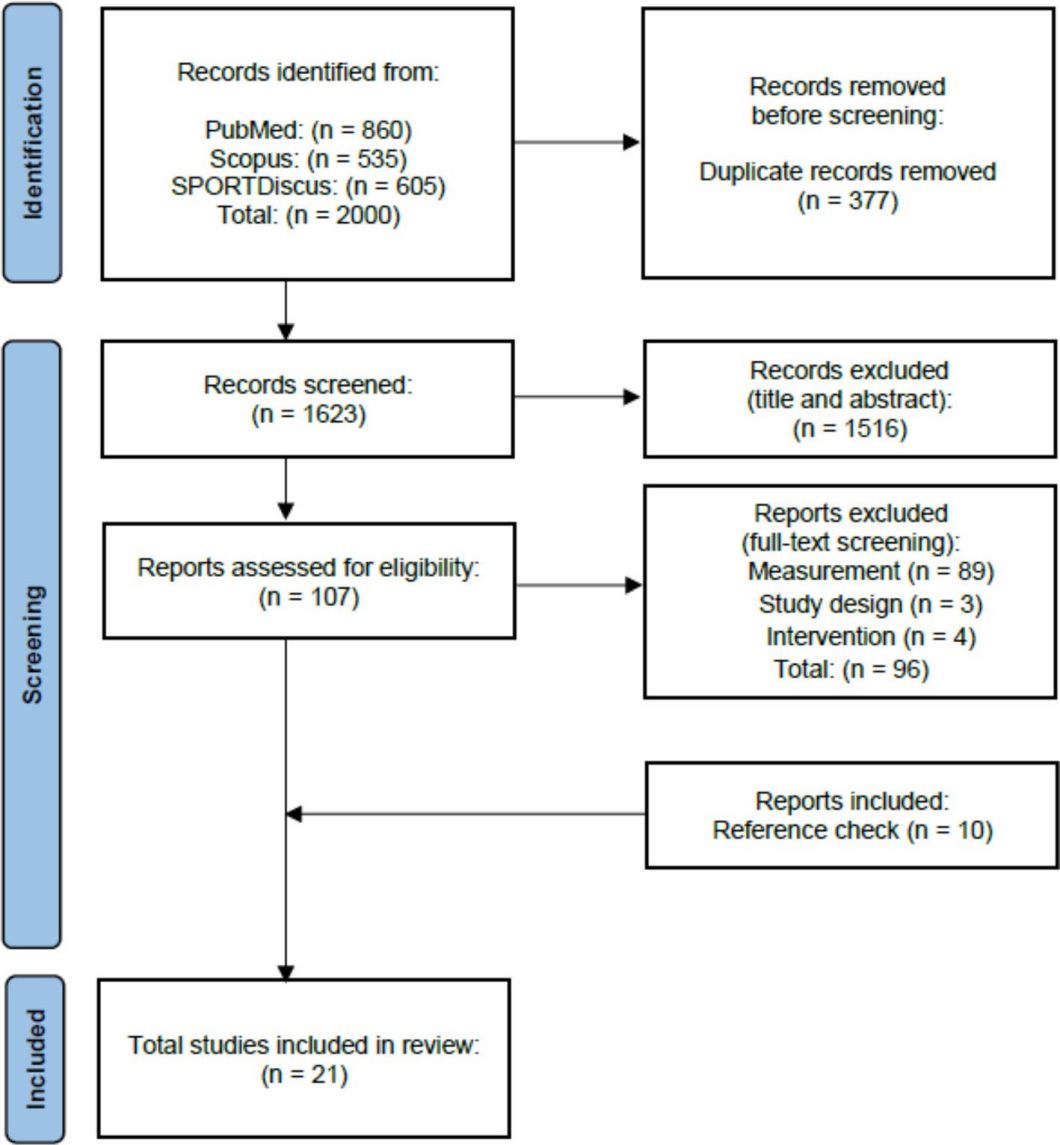
Study selection process as per the Preferred Reporting Items for Systematic reviews and Meta-Analyses extension for Scoping Reviews (PRISMA-ScR)

To identify relevant articles for the current scoping review, systematic literature searches were conducted from inception through April 2023, using PubMed, SPORTDiscus, and Scopus. Relevant studies were identified with the following search terms: “cross education” OR “cross transfer” OR “contralateral effect” OR “contralateral transfer” OR “interlimb transfer” OR “bilateral transfer” AND “endurance.” An additional search was carried out by examining the references of the included articles. Following the removal of duplicates, articles were screened first by title and abstract, followed by full text screening for eligibility. The study selection process is summarized using the PRISMA flow diagram (Fig. 1). In the present scoping review, broad inclusion criteria were used to provide an overview of the existing literature on cross-education of muscular endurance. To be included within the scoping review, studies were required to fulfill the following criteria: (1) original article was written in English language; (2) included a unilateral resistance exercise training intervention (regardless of strength training type and training load); (3) measured muscular endurance (e.g., number of repetitions at an absolute or relative load, time to task failure, total work) in the contralateral untrained limb at pre- and posttesting; and (4) was performed in humans with no restrictions on age and training status. One reviewer (JSS) completed literature searches and extraction of data. The following information was extracted: characteristics of participants, unilateral resistance training intervention (exercise type, sets, repetitions, load), frequency, duration, and main outcomes (cross-education of strength and muscular endurance). Two reviewers (JSS and JPL) checked the studies that only reported within-group changes (i.e., pre- to posttest) for each group, and back-calculated the *p*-value of between-group differences when possible.

## Results

### Search Results

The systematic search provided 2000 articles (PubMed = 860, Scopus = 535, SPORTDiscus = 605), of which 377 were duplicates, leaving 1623 for screening. After title/abstract screening, 1516 articles were excluded and the remaining 107 articles were assessed for eligibility via full-text screening. Ninety-six articles were omitted following the full-text assessment, and 10 additional studies were included by reference checking. In total, 21 studies met the aforementioned criteria and were included in the review.

### Study Characteristics

The present review included both randomized controlled trials and nonrandomized controlled trials. Of the 21 articles included in the review (Table 1), 10 studies were randomized controlled trials (including a nontraining control group) [21–30] and 11 studies were nonrandomized controlled trials [31–41]. Of note, this review focused more on randomized controlled trials, as it allows determination of whether changes in muscular endurance in an untrained limb (i.e., cross-education of muscular endurance) are solely due to the training interventions.

**Table 1:** Studies of cross-education of muscular endurance

Study	Participant	Unilateral resistance training intervention (group)	Frequency (duration)	Findings (untrained limb)
Randomized controlled trials (RCTs)				
Fariñas et al. [21]	Young Adults	A. Knee extension (4 sets × 8 reps, 10RM load) B. Knee extension (32 reps × 17.4 s rest between, 10RM load) C. Control	2 sessions (× 5 weeks)	Endurance (AB, Reps, 10RM): A ≈ B ≈ C Strength (1RM): A > B ≈ C Strength (MVIC): A ≈ B ≈ C
Fariñas et al. [22]	Young Adults	A. Biceps curl (5 sets × 6 reps, 10RM load) B. Biceps curl (30 reps × 18.5 s rest between, 10RM load) C. Control	2 sessions (× 5 weeks)	Endurance (AB, Reps, 10RM): A ≈ B ≈ C Strength (1RM): A > B ≈ C Strength (MVIC): A ≈ B ≈ C
Ben Othman et al. [23]	Adolescent Males	A. Leg press (4–8 sets × 5RM) B. Leg press (1–2 sets × 20RM) C. Control	3 sessions (× 8 weeks)	Endurance (AB or RE, Reps, 60% 1RM): B > A > C Strength (1RM): A ≈ B > C
Kannus et al. [24]	23–40 years Adults	A. Isokinetic knee extension and flexion (5 sets × 10 maximal reps at 240°/s, 5 sets × 5 maximal reps at 60°/s, 5 sets × 25 maximal reps at 240°/s) + isometric knee extension (5 sets × 10 s maximal rep at a knee flexion angle of 60°, 5 sets × 10 s maximal rep at a knee flexion angle of 30°) B. Control	3 sessions (× 7 weeks)	Group A: Endurance (total work, isokinetic 240°/s): Pre < Post Endurance (work in last 5 reps, isokinetic 240°/s): Pre < Post Strength (KE, MVIC): Pre < Post Strength (KF, MVIC): Pre ≈ Post Strength (KE, isokinetic 60°/s): Pre < Post Strength (KF, isokinetic 60°/s): Pre ≈ Post Strength (KE, isokinetic 240°/s): Pre < Post Strength (KF, isokinetic 240°/s): Pre ≈ Post Group B: All variables: Pre ≈ Post
Shaver [25]	Young Males	A. Elbow flexion (1 set × 30 reps/min until failure with 9.1 kg) B. Elbow flexion (1 set × 30 reps/min until failure with 9.1 kg) C. Elbow flexion (1 set × 30 reps/min until failure with 9.1 kg) D. Control After 6 weeks of training intervention, each training group received 1, 3, or 5 weeks of detraining intervention	3 sessions (× 6 weeks)	Group A, B, C: Endurance (RE, Reps, 10% MVIC): Pre < Post Endurance (RE, Reps, 15% MVIC): Pre < Post Endurance (RE, Reps, 20% MVIC): Pre < Post Endurance (RE, Reps, 25% MVIC): Pre < Post Strength (MVIC): Pre < Post Group D: All variables: Pre ≈ Post
Shaver [26]	Young Males	A. Elbow flexion (1 set × 30 reps/min until failure with 9.1 kg) B. Elbow flexion (1 set × 30 reps/min until failure with 9.1 kg) C. Elbow flexion (1 set × 30 reps/min until failure with 9.1 kg) D. Control After 6 weeks of training intervention, each training group received 1, 3, or 5 weeks of detraining intervention	3 sessions (× 6 weeks)	Group A, B, C: Endurance (AB, Reps, 9.1 kg): Pre < Post Group D: Endurance (AB, Reps, 9.1 kg): Pre ≈ Post
Shaver [27]	Young Males	A. Elbow flexion (1 set × 10 reps with 50% of 10RM, 1 set × 10 reps with 75% of 10RM, 1 set × 10 reps with 10RM) B. Control	3 sessions (× 6 weeks)	Endurance (RE, Reps 20% MVIC): A > B Endurance (RE, Reps, 25% MVIC): A > B Endurance (RE, Reps, 30% MVIC): A > B Endurance (RE, Reps, 35% MVIC): A > B Strength (MVIC): A > B
Meyers [28]	Young Males	A. Isometric elbow flexion (3 sets × 6 s maximal rep at an elbow flexion angle of 170°) B. Isometric elbow flexion (20 sets × 6 s maximal rep at an elbow flexion angle of 170°) C. Control	3 sessions (× 6 weeks)	Endurance (TTF, 100% MVIC): A ≈ B ≈ C Strength (MVIC 170°): A ≈ B ≈ C Strength (MVIC 90°): A ≈ B ≈ C
Kruse and Mathews [29]	Young Males	A. Elbow flexion (1 set × 30 reps/min until failure with 3/8 of maximum strength, 2 sessions/week) B. Elbow flexion (1 set × 30 reps/min until failure with 3/8 of maximum strength, 3 sessions/week) C. Elbow flexion (1 set × 30 reps/min until failure with 3/8 of maximum strength, 4 sessions/week) D. Elbow flexion (1 set × 30 reps/min until failure with 3/8 of maximum strength, 5 sessions/week) E. Control	2–5 sessions (× 4 weeks)	Group A, B, C, D, E: Endurance (AB or RE, Reps, 3/8 MVIC): Pre ≈ Post Strength (MVIC): Pre ≈ Post
Slater-Hammel [30]	Young Males	A. Elbow flexion (1 set × 35 reps/min until failure with 6.4 kg) B. Control	3 sessions (× 3 weeks)	Endurance (AB, Reps, 6.4 kg): A > B Strength: not reported
Nonrandomized and/or uncontrolled trials				
Hedayatpour et al. [31]	Young Males	A. Leg press (3 sets × 15 reps, 60% 1RM)	3 sessions (× 12 weeks)	Endurance (RE, TTF, 50% MVIC): Pre < Post Strength: not reported
Yuza et al. [31]	Young Females	A. Handgrip exercise (1 set × 0.5 s on and 0.5 s off until failure, 1/3 of maximum handgrip strength)	5 sessions (× 4 weeks)	Endurance (AB or RE, Reps, 1/3 MVIC): Pre < Post Strength (MVIC): Pre ≈ Post
Pincivero et al. [33]	Young Adults	A. Isokinetic knee extension and flexion (4–8 sets × 10 maximal reps, 40 s rest between sets) B. Isokinetic knee extension and flexion (4–8 sets × 10 maximal reps, 160 s rest between sets)	3 sessions (× 4 weeks)	Group A: Endurance (total work in 30 reps, KE isokinetic 180°/s): Pre < Post Endurance (total work in 30 reps, KF isokinetic 180°/s): Pre ≈ Post Strength (KE, concentric 60°/s): Pre ≈ Post Strength (KF, concentric 60°/s): Pre ≈ Post Strength (KE, concentric 180°/s): Pre ≈ Post Strength (KF, concentric 180°/s): Pre > Post Group B: Endurance (total work in 30 reps, KE isokinetic 180°/s): Pre ≈ Post Endurance (total work in 30 reps, KF isokinetic 180°/s): Pre ≈ Post Strength (KE, concentric 60°/s): Pre ≈ Post Strength (KF, concentric 60°/s): Pre > Post Strength (KE, concentric 180°/s): Pre < Post Strength (KF, concentric 180°/s): Pre ≈ Post
Sinoway et al. [34]	Young Males	A. Handgrip exercise (1 set × 12 reps/min until failure, 30–35% MVC)	5 sessions (× 4 weeks)	Endurance (AB or RE, TTF, 70% of the highest sustainable 3 min workload): Pre < Post Strength (MVIC): Pre > Post
Grimby et al. [35]	Old Males	A. Isometric knee extension (2 sets × 2 maximal reps for 4 s at a knee flexion angle of 60°, 1 set × 2 maximal reps for 4 s at a knee flexion angle of 30°) + isokinetic concentric knee extension (1 set × 8 maximal reps at 30°/s, 1 set × 8 maximal reps at 180°/s) + isokinetic concentric/eccentric knee extension (3 sets × 8 maximal reps at 30°/s)	2–3 sessions (× 8–11 weeks)	Endurance (KE, difference in work from the first 3 reps to the last 3 reps during 50 reps): Pre ≈ Post Strength (KE, concentric 30°/s): Pre ≈ Post Strength (KE, eccentric 30°/s): Pre ≈ Post Strength (KE, concentric 120°/s): Pre ≈ Post Strength (KE, eccentric 120°/s): Pre ≈ Post
Parker [36]	Young Males	A. Isometric knee extension (1 set × 10–30 brief maximal reps at a knee flexion angle of 90°) B. Dynamic knee extension (1 set × 100–300 reps with 6.4 kg)	3–6 sessions (× 4 months)	Group A: Endurance (AB or RE, TTF, 60% MVIC): Pre ≈ Post Strength (MVIC): Pre < Post Group B: Endurance (AB or RE, TTF, 60% MVIC): Pre ≈ Post Strength (MVIC): Pre ≈ Post
Tesch and Karlsson [37]	Young Males	A. Isometric leg press (3 sets × sustained contraction at 50% MVIC until failure)	3–4 sessions (× 6 weeks)	Endurance (AB or RE, TTF, 50% MVIC): Pre < Post Strength (MVIC): Pre ≈ Post
Yasuda and Miyamura [38]	Young Males	A. Handgrip exercise (1 set × 60 reps/min until failure with 1/3 maximum grip strength) B. Handgrip exercise (1 set × 60 reps/min until failure with 1/2 maximum grip strength)	6 sessions (× 6 weeks)	Group A: Endurance (AB or RE, Reps, 1/3 MVIC): Pre ≈ Post Strength (MVIC): Pre ≈ Post Group B: Endurance (AB or RE, Reps, 1/2 MVIC): Pre < Post Strength (MVIC): Pre < Post
Hodgkins [39]	Young Females	A. Knee extension (1 set × 10 reps/min until failure with a 8.2 kg boot)	3 sessions (× 3 weeks)	Endurance (AB, Reps, 8.2 kg): Pre < Post Strength: not reported
Walters et al. [40]	Young Adults	A. Isometric elbow flexion (3 sets × 15 s maximal rep) B. Isometric elbow flexion (3 sets × 15 s rep at 2/3 of maximum strength) C. Isotonic elbow flexion (3 sets × as many repetitions as possible within 15 s, intensity/load not provided)	3–5 sessions (× 2 weeks)	Group A: Endurance (AB or RE, Reps, 1/3 1RM): Pre ≈ Post Strength (MVIC): Pre < Post Group B: Endurance (AB or RE, Reps, 1/3 1RM): Pre ≈ Post Strength (MVIC): Pre ≈ Post Group C: Endurance (AB or RE, Reps, 1/3 1RM): Pre ≈ Post Strength (MVIC): Pre ≈ Post
Mathews et al. [41]	Young Males	A. Elbow flexion (1 set × 30 reps/min until failure) Strength test for elbow flexion was also performed during each session (no detail provided)	3 sessions (× 4 weeks)	Endurance (AB, Reps, 3/8 MVIC): Pre ≈ Post Strength (EF, MVIC): Pre < Post

*AB*: absolute muscular endurance; *AB or RE*: the study did not clearly indicate whether and absolute or relative muscular endurance test was used; *KE*: knee extension; *MVIC*: maximum voluntary isometric contraction; *RE*: relative muscular endurance; *Reps*: maximal number of repetitions; *TTF*: time to task failure; *1RM*: one-repetition maximum; > significant difference between groups (e.g., A > B indicates that group A had greater changes in muscular endurance in the untrained limb compared to group B); *≈*: no significant difference between groups (e.g., A ≈ B indicates that the changes in muscular endurance in the untrained limb were not different between group A and B)

Among the 21 included studies, nine studies employed unilateral exercise training in the lower body (3 randomized controlled trials and 6 nonrandomized controlled trials) [21, 23, 24, 31, 33, 35–37, 39], nine studies in the upper arm (7 randomized controlled trials and 2 nonrandomized controlled trials) [22, 25–30, 40, 41], and three studies used handgrip (3 nonrandomized controlled trials) [32, 34, 38]. For the muscular endurance measurements, absolute muscular endurance (i.e., number of repetitions with the same given load at pre- and postintervention, regardless of changes in maximal strength) was assessed in six studies [21, 22, 25, 30, 39, 41], and relative muscular endurance (i.e., number of repetitions with a load corresponding to a specific relative intensity or percentage of individual’s current 1RM) was measured in two studies [26, 27]. Of note, five studies did not provide enough detail to determine whether absolute or relative muscular endurance was examined for testing [23, 29, 32, 38, 40]. The remaining eight studies used several different types of muscular endurance measurements including: time to task failure (using absolute or relative load) [28, 31, 34, 36, 37], total work performed [24, 33], and fatigue index (e.g., difference in work between the first three reps and the last three reps) [35]. Among the 21 included studies, five studies were conducted in untrained individuals [23, 24, 31, 33, 38], whereas the remaining 16 studies did not clearly describe the training status of the participants (e.g., physically active, college students from physical education program) [21, 22, 25–30, 32, 34–37, 39–41].

## Discussion

### Findings from Nonrandomized Controlled Trials

Several nonrandomized controlled trials reported changes in muscular endurance in the untrained limb following unilateral exercise training interventions. For example, 3 weeks of unilateral knee extension training increased absolute muscular endurance (i.e., maximal number of repetitions using 8.2 kg) in the contralateral untrained leg from pre- to posttest [39]. Similarly, 12 weeks of unilateral leg press exercise training increased relative muscular endurance [i.e., time to task failure during sustained isometric knee extension at relative 50% maximum voluntary isometric contraction (MVIC)] in the untrained leg from pre- to posttest [31]. In addition, four studies observed an increased muscular endurance (i.e., maximal number of repetitions and time to task failure) in the untrained limb (i.e., pre- to posttest) following 4–6 weeks of unilateral handgrip exercise training [32, 34, 38] and 6 weeks of unilateral isometric leg press training [37]. In those studies, however, it was unclear whether they used an absolute or relative muscular endurance test [32, 34, 37, 38]. In one study, an increase in total work (i.e., during 30 maximum isokinetic knee extension) from pre- to posttest was observed in the untrained leg following 4 weeks of unilateral isokinetic knee extension and flexion [33]. However, these findings were not consistent throughout the literature. For example, no changes (i.e., pre- to posttest) in muscular endurance (i.e., absolute and/or relative, fatigue index) were observed in the contralateral untrained limb following unilateral knee extension training interventions [35, 36], or following unilateral elbow flexion training interventions [40, 41]. Of note, however, these findings should be interpreted with caution as it is not possible to know whether the changes in muscular endurance are due to the exercise training intervention or other factors outside of the training intervention. In other words, to determine whether the cross-education of muscular endurance is solely due to the training interventions, a time-matched nontraining control group is required (i.e., randomized controlled trials).

### Findings from Randomized Controlled Trials

Among ten randomized controlled trials [21–30], three studies reported a cross-education of muscular endurance [23, 30]. In male children (aged 10–13 years), for example, 8 weeks of unilateral leg press training increased not only strength but also muscular endurance (i.e., number of unilateral leg press repetitions with 60% of 1RM until failure) of the contralateral untrained leg compared with a nontraining control group [23]. In that study, however, it was not clear whether 60% of pre- or posttraining 1RM was used at the posttesting (i.e., absolute or relative muscular endurance) [23]. In healthy young males, 3 weeks of unilateral elbow flexion exercise training increased absolute muscular endurance (i.e., maximal number of unilateral elbow flexion repetitions with 6.4 kg) in the contralateral untrained arm compared with a nontraining control group [30]. In five randomized controlled trials, only within-group changes (i.e., pre- to posttest) in muscular endurance were reported [24–27, 29]. For example, increases in muscular endurance (i.e., total work performed during 25 maximal isokinetic contractions and work performed during the last 5 repetitions) were observed in the contralateral untrained leg from pre- to posttest in a group that performed 7 weeks of isokinetic and isometric knee extension training, whereas no within-group changes were observed in a time-matched nontraining control group [24]. Similarly, increases in absolute and relative [25, 26] muscular endurance from pre- to posttest were observed in the untrained arm following 6 weeks of unilateral elbow flexion training, while no changes were observed in a nontraining control group. In contrast, one study found no within-group changes (pre- to posttest) in either the training (i.e., 4 weeks of unilateral elbow flexion training) group or the nontraining control group [29]. Although some studies reported increases in muscular endurance only in the training groups and not in the control groups, this does not indicate that there was cross-education of muscular endurance. To determine whether a cross-education of muscular endurance is present, the changes in muscular endurance of the training groups should be directly compared with those of the control group. In one study, although only within-group changes (i.e., pre- to posttest) were reported for training and control groups, we were able to directly compare those two groups by back-calculating the *p*-value of between-group differences [27]. The calculation showed that the changes in relative muscular endurance in the untrained arm following 6 weeks of unilateral elbow flexion training were significantly greater compared with a control group, indicating a cross-education of relative muscular endurance [27]. Three randomized controlled trials did not observe cross-education of muscular endurance [21, 22, 28]. For example, no changes in absolute muscular endurance were observed in the contralateral untrained limb following 5 weeks of unilateral knee extension training [21] and following 5 weeks of unilateral elbow flexion training [22] when compared with a nontraining control group. Similarly, no changes in time to failure (i.e., sustaining at 100% MVIC until force drop below 50% MVIC) were observed in the untrained arm following 6 weeks of unilateral isometric elbow flexion training when compared with a nontraining control group [28].

Taken together, there is very limited evidence to suggest that performing unilateral resistance training can increase muscular endurance in the contralateral untrained limb (i.e., cross-education of muscular endurance). For example, there have been only three randomized controlled studies (out of 10 studies) that demonstrated evidence for cross-education of muscular endurance. Among these three studies, one showed increased absolute muscular endurance, another showed increased relative muscular endurance, and the third study showed increased muscular endurance (unclear whether absolute or relative). In contrast, the remaining seven studies either did not find or could not provide supporting evidence. These discrepancies in the cross-education of muscular endurance may be due to the differences in training interventions (e.g., contraction type, intensity, duration) and/or muscular endurance measurements (e.g., maximal number of repetitions and time to task failure using absolute or relative load). The current body of literature does not provide sufficient evidence to draw a clear conclusion on whether cross-education of muscular endurance is present, and thus requires further investigation.

### Potential Underlying Mechanisms

There have been several mechanisms proposed to explain the increase in muscular endurance in the trained limb following resistance training, such as increased muscle capillarity [17] and mitochondrial respiratory capacity/function [42, 43]. Although these proposed mechanisms may explain training-induced increases in muscular endurance in the trained limb, these would be unlikely to explain the changes in the contralateral untrained limb. The following section will discuss potential mechanisms that might contribute to the cross-education of muscular endurance.

#### Increases in Muscle Strength (Cross-Education of Strength)

One potential adaptation that could improve absolute muscular endurance in the contralateral untrained limb following unilateral resistance training is increased strength in the untrained limb via cross-education (i.e., cross-education of strength). According to the size principle, motor units are recruited in an orderly manner from the smaller motor units (i.e., low threshold) to the larger motor units (i.e., high threshold) as required force increases or muscle fatigues [44]. Based on this, increases in strength following resistance training may require fewer motor units to lift an absolute submaximal load for the same number of repetitions, which may delay the involvement of larger motor units and reserve them to be recruited subsequently for sustaining the required force as fatigue develops [14, 45]. This hypothesis is partially supported by Ploutz et al. [45] who showed that less muscle was recruited to lift the same submaximal load in the untrained leg following 9 weeks of unilateral knee extension training [45], which may reserve larger motor units to be recruited later on and consequently allow for better performance on the absolute muscular endurance test in the untrained limb. However, this should be interpreted with caution since there was no time-matched control group, which makes it difficult to know whether the changes in muscle recruitment in the untrained limb were due to the unilateral training or some other factor [45]. The potential role of changes in strength on absolute muscular endurance may be also partially supported by a secondary analysis that examined if the changes in 1RM strength mediate changes in absolute muscular endurance (i.e., maximal number of repetitions using 42.5% pretraining 1RM) following high-load (i.e., 70% 1RM) training compared with low-load training interventions (i.e., 15% 1RM with or without blood flow restriction) [18]. In that study, it was found that training-induced increases in strength mediated the changes in muscular endurance in the high-load training group relative to the low-load training groups, suggesting that the differences in muscular endurance between high-load and low-load training groups may be explained by changes in strength. However, it is of note that the mediation analysis in that study only compared between training groups and not with a time-matched control group, meaning that the results can only explain the differences between training groups (i.e., high load versus low load). To clearly demonstrate whether the change in strength is an underlying mechanism for changes in muscular endurance, it may be more appropriate to compare training groups to a nonexercise control group in the mediation analysis. Furthermore, that analysis was on the changes in the trained limb, and thus it remains unknown whether increased strength from cross-education can also be translated to improved absolute muscular endurance in the untrained limb. One of the included studies reported concurrent increases in strength and muscular endurance in the untrained limb [23], whereas other studies showed that the cross-education of strength is not always accompanied by the cross-education of absolute muscular endurance [21, 22]. Of note, simply assessing whether there were concurrent cross-education of strength and absolute muscular endurance may not be an appropriate approach to determine whether cross-education of strength can be translated to cross-education of absolute muscular endurance. A more appropriate approach might be using a mediation analysis to examine if the increases in strength from cross-education mediate the changes in absolute muscular endurance in the untrained limb [46, 47]. It is of note that some previous studies have shown that unilateral low-load (or low-intensity) training does not increase strength in the opposite untrained limb (i.e., no cross-education of strength) [48, 49]. However, this does not necessarily mean that unilateral low-load (or low-intensity) exercise would not induce cross-education of muscular endurance. It is plausible that cross-education of muscular endurance can occur in the absence of strength gain via different mechanisms.

#### Bilateral Access and Cross-Activation Model

Cross-education of relative muscular endurance likely cannot be explained by increased strength in the contralateral limb as relative muscular endurance is scaled to current maximal strength. Two main theoretical models, which may not be mutually exclusive, have been proposed to explain the cross-education of strength and skills: “bilateral access” and “cross-activation” models [50]. Although speculative, these two models may also explain the cross-education of muscular endurance. The “bilateral access” model involves the development of a motor engram during unilateral resistance training, which can be accessed not only by the trained limb, but also by the untrained limb for the control and execution of movements [50, 51]. A widely used example is the “callosal access” hypothesis, in which the motor engrams developed in the trained hemisphere may be accessed by the opposite untrained hemisphere via the corpus callosum during motor tasks in the untrained limb [50, 51]. In this model, it has been hypothesized that performing unilateral resistance training may develop an effective muscle recruitment pattern for maximum force production (i.e., muscle strength), such as coordination of synergists and inhibition of antagonists, which can be stored in neural circuits and accessed by the untrained hemisphere [4]. Although speculative, this hypothetical model may also play a role in the cross-education of muscular endurance. In other words, performing unilateral resistance training may create a motor engram of the motor output necessary to effectively perform repeated submaximal contractions, leading to cross-education of muscular endurance. However, further research is needed to determine whether or not the “bilateral access” model plays a role in the cross-education of muscular endurance in a similar way as cross-education of strength. In the “cross-activation” model, it is proposed that performing unilateral resistance training could induce bilateral cortical activation, potentially leading to concurrent neural adaptations in both trained and untrained hemispheres [50, 52–55]. For example, it was previously found that unilateral resistance training increased corticospinal excitability in both the trained and untrained primary motor cortex [55]. Furthermore, decreases in interhemispheric inhibition [56], short-interval intracortical inhibition [52, 57], and cortical silent period [58, 59] were also observed in both the trained and untrained side following unilateral resistance training. However, whether or not these neural adaptations can explain the cross-education of muscular endurance is currently not known, and further research is needed.

#### Increase in Tolerance to Exercise-Induced Discomfort

Increases in tolerance to exercise-induced discomfort may in part play a role in the cross-education of muscular endurance. For example, previous studies have suggested that the cross-education of muscular endurance may be due to repeated exposures to uncomfortable exertions during a training intervention, which allows individuals to accommodate greater exercise-induced discomfort, pain, and/or fatigue sensation [23, 27, 30, 32]. Although it is not directly related to exercise-induced discomfort perception, previous cross-sectional studies have demonstrated that athletes typically have higher pain tolerance when compared with nonathlete control individuals [60, 61]. In addition, increased pain tolerance has been observed following aerobic and combined (aerobic + resistance) exercise training in healthy young adults [62]. It has been proposed that the higher pain tolerance observed in trained individuals may be due to enhanced pain coping strategies, developed through repeated exposure to physical and psychological stress during exercise [60, 63]. This is further supported by a previous study in which 6 weeks of high-intensity interval training increased not only ischemic pain tolerance but also exercise tolerance (i.e., time to exhaustion) when compared with volume-matched moderate-intensity continuous training [64]. In that study, it was suggested that the improvement in pain tolerance is likely due to repeated exposure to high metabolic stress and exercise-induced noxious stimuli, which might partly explain the improvement in exercise tolerance [64]. Based on these findings, it is possible that repeated exposure to discomfort from unilateral resistance training can lead to increased tolerance, resulting in increased muscular endurance in the contralateral untrained limb. This proposed mechanism is unlikely to play a role in the cross-education of muscular endurance if the training intervention only induces very low levels of discomfort or pain (e.g., low repetition with low load). However, since this proposed mechanism is based on a study that implemented aerobic training intervention, it needs to be further examined with resistance training intervention.

### Future Considerations

There has been extensive work on the cross-education of strength, but far less attention has been paid to the cross-education of muscular endurance. For example, there is a lack of randomized controlled studies, which makes it difficult to draw clear conclusions on the cross-education of muscular endurance. The inclusion of a time-matched nonexercise control group allows researchers to confidently conclude that the increase in muscular endurance in the untrained limb is due to the unilateral resistance training and not to some other factor. Thus, time-matched control groups are always recommended for future studies. In addition, it is common to see studies reporting within-group changes (i.e., pre- to posttest) for each training and control group, and when significant changes are observed only in the training group and not in the control group, it is often concluded that there is cross-education of muscular endurance. However, this interpretation is problematic since the change scores are not directly compared between groups (e.g., intervention group versus control group). In other words, it is important to test the group × time interaction or directly compare the change scores between the groups if the goal is to examine whether the changes in muscular endurance in the untrained limb differ between the groups [65–67].

Several included studies in the present review did not clearly indicate how they measured the cross-education of muscular endurance. For example, a number of studies measured the maximal number of repetitions using a certain percentage of maximum strength (e.g., 30% of 1RM); however, they did not clearly indicate whether an absolute or relative load/intensity was utilized at posttesting. This lack of clarity makes it difficult to compare results across the literature and to replicate the data in future works. Therefore, future studies should clearly state within their methodology whether muscular endurance was measured via an absolute or relative muscular endurance test. In addition to absolute/relative muscular endurance, several other types of outcome variables have been also examined to test muscular endurance (e.g., total work during a certain number of repetitions, time to task failure). This discrepancy in methodology may partially explain the inconsistent findings observed in the existing literature. At present, it remains unclear which outcome variable is the most appropriate way to test an individual’s muscular endurance, and thus further research is warranted. Of note, in the cross-education of strength literature, it has been suggested that the changes in strength in the contralateral untrained limb are the greatest when it is tested with the same movement task performed by the trained limb (training specificity; e.g., test and train dynamically) [4]. Based on this, it may be reasonable to test the cross-education of muscular endurance with the same movement task used for the training intervention. However, the question of whether the cross-education of muscular endurance follows the principle of specificity requires further investigation.

Future studies might examine other markers of endurance capacity (e.g., mitochondrial density, muscle capillarization) to provide better support for the idea that the mechanism underlying cross-education of muscular endurance may not be local per se, but potentially via neural adaptations. A final consideration for future studies, especially for those attempting to address potential underlying mechanisms, may be the use of mediation analysis. In the present review, we suggested a number of potential underlying mechanisms including changes in strength in the untrained limb (for absolute muscular endurance). In one of the included studies, for example, concurrent increases in strength and muscular endurance were observed in the untrained limb (i.e., cross-education of strength and muscular endurance) [23]. However, because there was concurrent cross-education of strength and absolute muscular endurance, this does not necessarily indicate that the cross-education of muscular endurance was driven by the cross-education of strength. One statistical approach to understanding the potential role of strength changes in cross-education of muscular endurance may be using a mediation analysis [46, 47]. Mediation analysis can quantify the effect of the third (mediating) variable (e.g., changes in strength in untrained limb) on the relationship between the independent variable (e.g., intervention groups) and dependent variable (e.g., changes in absolute muscular endurance in untrained limb). This approach may help future studies with identifying the potential underlying mechanisms that contribute to the cross-education of muscular endurance (if it exists).

## Conclusions

Performing unilateral resistance training has been shown to increase strength not only in the trained limb but also in the contralateral untrained limb (i.e., cross-education of strength). However, less attention has been paid to the question of whether performing unilateral resistance training can also increase muscular endurance in the contralateral untrained limb (i.e., cross-education of muscular endurance). The current body of the literature does not provide sufficient evidence to draw clear conclusions on whether a cross-education of muscular endurance is present. Therefore, further research with a nonexercise control group (i.e., randomized controlled trials) is necessary to draw a strong conclusion. Furthermore, some potential underlying mechanisms (i.e., increased strength, bilateral access model, increased tolerance) are discussed in the present review; however, the proposed ideas currently lack experimental evidence and require further research.

## Declarations

### Availability of Data and Material

Available upon request from the corresponding author.
